# Examination of brain area volumes based on voxel-based morphometry and multidomain cognitive impairment in asymptomatic unilateral carotid artery stenosis

**DOI:** 10.3389/fnagi.2023.1128380

**Published:** 2023-03-15

**Authors:** Wei Duan, Li Lu, Chun Cui, Tongsheng Shu, Dazhi Duan

**Affiliations:** ^1^Department of Neurology, The Second Affiliated Hospital (Xinqiao Hospital), Army Medical University (Third Military Medical University), Chongqing, China; ^2^Department of Radiology, The Second Affiliated Hospital (Xinqiao Hospital), Army Medical University (Third Military Medical University), Chongqing, China

**Keywords:** carotid artery stenosis, cognitive impairment, gray matter, white matter, voxel-based morphometry analysis

## Abstract

**Objective:**

Recent evidence has demonstrated that unilateral carotid artery stenosis (CAS) can contribute to the development of cognitive impairment. However, the features of cognitive dysfunction induced by unilateral CAS remain unclear.

**Methods:**

Sixty asymptomatic patients with unilateral CAS were divided into mild, moderate and severe stenosis groups. These patients and 20 healthy controls provided clinical data and serum, which was used to assess the levels of certain vascular risk factors. Then, they participated in a battery of neuropsychological tests. Additionally, all participants underwent a 3.0 T magnetic resonance imaging (MRI) scan of the brain. Chi-square tests and one-way ANOVA were used to determine significant differences in the risk factors and cognitive test scores between groups. Multiple logistic regression analysis and the receiver operating characteristic (ROC) curve analysis were performed to identify the independent risk factors for cognitive impairment in patients with CAS. Finally, fluid attenuated inversion recovery (FLAIR) T1-weighted MRI images were processed by voxel-based morphometry (VBM) analysis using the Statistical Parametric Mapping (SPM) 8 software.

**Results:**

Compared with healthy controls, the scores of the Mini-Mental State Examination, Digital Span Test backward, and Rapid Verbal Retrieve were significantly reduced in patients with left CAS. The scores in all cognitive scales were significantly lower in patients with right CAS than in controls. Logistic regression analysis demonstrated that the degree of carotid stenosis was an independent risk factor for cognitive impairment in asymptomatic patients with unilateral CAS. Furthermore, VBM analysis showed that, compared with those in healthy controls, gray matter and white matter volumes in specific brain areas were markedly decreased in patients with severe unilateral CAS. However, in patients with moderate right CAS, there was a significant decline in the volume of gray matter in the left parahippocampal gyrus and supplementary motor area. Additionally, the volume of white matter in the left insula was obviously lower in patients with moderate right CAS than in healthy controls.

**Conclusion:**

Unilateral asymptomatic CAS, especially on the right side, contributed to cognitive impairment, including memory, language, attention, executive function and visuospatial function. In addition, based on VBM analysis, both gray matter atrophy and white matter lesions were found in patients with unilateral asymptomatic CAS.

## Introduction

Cognitive impairment affects the patients’ well-being and their ability to live independent and productive lives (Chaytor and Schmitter-Edgecombe, 2003). It also places large demands on societal, hospital, and financial resources (Rockwood et al., 2002). Although, the prevalence of vascular cognitive impairment induced by ischemic or hemorrhagic stroke or transient ischemic attack increases gradually with age, increasing evidence suggests that cognitive impairment also occurs in patients with asymptomatic carotid artery stenosis (CAS) (Xiang et al., 2013; Lal et al., 2017; Pillai, 2022). Atherosclerosis is a direct consequence of multiple vascular risk factors and can lead to increased intima-media thickness (IMT), plaque formation, and subsequent carotid artery stenosis or occlusion (Xiang et al., 2013). An increasing number of studies have demonstrated that CAS induced by atherosclerosis might be a potential risk factor for cognitive impairment in elderly people. Recent data has further indicated that patients with unilateral CAS might present with specific cognitive impairment relevant to the ipsilateral hemispheric functions (Huang et al., 2014). Moreover, high-grade stenosis of the unilateral carotid artery might contribute to multidomain cognitive impairment (Yue et al., 2016; He et al., 2021). However, there is limited knowledge on the characteristics and differences in cognitive impairment caused by unilateral CAS. Furthermore, the underlying mechanism of cognitive dysfunction induced by CAS remains unclear.

In the present study, we enrolled asymptomatic patients with three different levels of unilateral CAS as well as age-, sex-, and education level-matched healthy controls. A comprehensive battery of standardized neuropsychological tests was performed to analyze the cognitive impairment of the four groups. Additionally, we compared clinical vascular risk factors between the patients with unilateral CAS and healthy controls and determined independent risk factors for cognitive impairment. Finally, fluid-attenuated inversion recovery (FLAIR) T1-weighted magnetic resonance imaging (MRI) images were acquired, and voxel-based morphometry (VBM) analysis (Takeuchi and Kawashima, 2017) using statistical parametric mapping (SPM) was performed to explore the abnormal gray and white matter volumes in asymptomatic patients with unilateral CAS.

## Materials and methods

### Subjects

Patients with different levels of unilateral carotid artery stenosis (n = 60) and healthy controls (n = 20) were recruited to participate in this study between June 2017 and January 2021 in the Department of Neurology in Xinqiao Hospital, Army Military Medical University. All patients underwent head and neck computed tomography angiography (CTA) to identify the degree of their carotid artery stenosis. The enrolled patients with carotid artery stenosis were divided into three groups by 2 neurologists and 1 radiologist: mild, moderate and severe. The degree of CAS was also classified according to the North American Symptomatic Carotid Endarterectomy Trial (NASCET) standard into categories of no stenosis, mild stenosis (0–29%), moderate stenosis (30–69%), severe stenosis (70–89%), subtotal occlusion and occlusion. It should be noted that patients with previous stroke history or transient ischemic attack in either hemisphere or other diseases or medical events, such as intracranial tumors, central nervous system demyelinating diseases, severe brain trauma, history of neurological surgery, infection, or carbon monoxide poisoning, were excluded from the study. We also excluded patients with severe hearing and visual impairment, precluding a reliable assessment of cognitive function, or with severe psychotic disorders. Finally, patients with carotid revascularization on either side, contralateral arterial occlusion, known vertebral basilar or intracranial stenosis or occlusion, intracranial arteriovenous malformation, or documented dementia were excluded. Patients with carotid artery stenosis did not have ischemic or hemorrhagic lesions in the brain, and healthy controls had no stenosis in the bilateral carotid artery detected by head and neck CTA. This study was approved by the medical ethics committee of Xinqiao Hospital, Army Military Medical University, Chongqing, China. Full written informed consent for participation was obtained from each subject.

During admission, all participants provided demographic data and medical history. Age, sex, education, high blood pressure (HBP) and diabetes mellitus (DM) history, current smoking (CS) and current alcohol use (CAU) were obtained from the patient-administered questionnaire. Total cholesterol (TC), triglyceride (TG), high-density lipoprotein cholesterol (HDL-C), low-density lipoprotein cholesterol (LDL-C), and uric acid (UA) were also measured.

### Neuropsychological evaluation

The battery of neuropsychological tests assessing a range of cognitive domains comprised the Chinese version of the Mini-Mental State Examination (MMSE), Trail-Making Test (TMT), Digital Span Test (DST), Rapid Verbal Retrieve (RVR), and Clock Drawing Test (CDT). All participants were administered the full cognitive battery. Testing was conducted in a quiet room and administered by a master’s-level neuropsychology technician blinded to study participant status (stenosis vs. control) under the supervision of a senior neuropsychologist. The order of administration was consistent across participants. The total testing time varied modestly among participants, ranging from 70 to 90 min, because of interindividual variability in completion time for tests without specific time limits. The tests were scored by a single neuropsychologist. Because mood could also influence results, the Hamilton Depression Scale (HAMD) was administered concurrently.

Overall cognitive functions were detected in the MMSE. The MMSE was developed from different neuropsychological batteries and includes five sections worth a total of 30 points: orientation (10 points); registration (3 points); attention and calculation (5 points); recall (3 points); and language (9 points). A score of less than 24 points indicated cognition impairment. The TMT score reflected the information processing speed of the participants. In the TMT, patients were required to quickly draw lines to connect consecutively numbered circles in ascending order. The maximum test duration was set at 300 s, and the number of errors, as well as the time taken to complete the test (measured in seconds), were recorded. Any patient who stopped midway was asked to continue the test for the remainder of the test duration. If not completed within the time limit, an error was recorded. The DST score reflected the concentration, instantaneous memory, and resistance to information interference of the participants. These included forward and backward count tests, where an evaluator read a series of numbers aloud to the patients. Patients were then required to repeat these numbers in the same order that they heard or in the reverse order. One point was awarded for each test passed, and zero points were awarded for a failed test. The highest scores in the forward count test and backward count test were 16 and 14 points, respectively. Therefore, the highest total score was 30 points. The RVR score reflected the language and executive function of the participants. During the RVR, the patients were required to say as many animal names (such as cattle, horses, and sheep) as possible within 1 min and were scored based on the number of correct animal names. The patients were then asked to complete two more sections, listing the names of fruits and vegetables within 1 min each. The sum of the three scores was the total score of this test. The executive and visuospatial functions were examined in the CDT. The CDT required the patients to draw a clock dial on white paper independently, place the 12 numbers in the correct positions, and mark the position of the specified time with a watch needle. The most common and simple scoring method of CDT was the quartering method: (1) draw a closed circle, 1 point; (2) put the numbers in the correct positions, 1 point; (3) the dial includes all 12 correct figures, 1 point; and (4) place the pointer in the correct position.

### Magnetic resonance imaging protocols

MRI scanning of the brain was performed using a 3.0 Tesla scanner (General Electric, Milwaukee, WI, United States) with a 12-channel head coil. Fast spin-echo (FSE) T2-weighted images and FLAIR T1-weighted images were acquired with TE/TR = 112.2/3160 ms and TE/IT/TR = 27.072/860/1696.68 ms. All MRI images were acquired with a voxel size of 0.4688 × 0.4688 × 5 mm (Lal et al., 2017), 20 sagittal slices and an in-plane resolution of 512 × 512.

### Data analysis

All statistical analyses of demographic and clinical variables were performed using SPSS version 20.0 (IBM SPSS, Chicago, IL, United States), and a value of *p* < 0.05 was considered statistically significant. Continuous variables are presented as their means ± standard deviations. Categorical variables are presented as percentages. Chi-square tests and one-way ANOVA were used to determine significant differences in the frequencies of categories and differences in continuous variables between the groups, respectively. The Pearson correlation coefficient was used to evaluate the correlation between vascular risk factors and declined cognitive functions. Multiple logistic regression analysis and receiver operating characteristic (ROC) curve analysis were performed to identify the independent risk factors for cognitive impairment in patients with carotid artery stenosis, with the degree of stenosis as a categorical variable.

The MRI data were processed using Statistical Parametric Mapping (SPM) 8 software (Wellcome Centre for Human Neuroimaging, UCL Queen Square Institute of Neurology, London, United Kingdom) with VBM implemented in the VBM 8 toolbox^1^ with default parameters (Duan et al., 2016). Images were bias-corrected, tissue classified, and registered using linear (12-parameter affine) and nonlinear transformations (warping) within a unified model. Subsequently, VBM analysis was performed on the normalized gray matter and white matter segments, which were multiplied by the nonlinear components derived from the normalization matrix to preserve actual gray matter and white matter values locally (modulated GM and WM volumes). Importantly, the segments were not multiplied by the linear components of the registration to account for individual differences in brain orientation, alignment, and size globally. Finally, the images were smoothed with a Gaussian kernel of 8 mm full width at half maximum (FWHM). Voxelwise differences in gray matter and white matter between each group were examined using independent-sample *t-test*s. To avoid possible edge effects between different tissue types, we excluded all voxels with gray matter and white matter values of less than 0.09 (absolute threshold masking). We also applied a threshold of *p* < 0.05 with 10-voxel clustering criteria.

## Results

### Clinical characteristics and cognitive assessments

Table 1 shows a comparison of clinical data between the patients with left carotid artery stenosis and healthy controls. There was a significant difference in smoking status, alcohol consumption and high blood pressure among the groups with different levels of carotid artery stenosis and the healthy control group (*p* < 0.05). Additionally, marked differences in the levels of TC and HCY are also shown in Table 1. As shown in Table 1, there were significant differences in the MMSE, DST (backward) and RVR scores of patients and healthy controls (*p*<0.05). Obvious differences were not present in the scores of the HAMD, TMT, DST (forward) and CDT. These data indicated that instantaneous memory and language function significantly declined in asymptomatic patients with left CAS.

**Table 1:** Differences of clinical risk factors and neuropsychological examinations of patients with left carotid artery stenosis.

Characteristic	Patients with left carotid artery stenosis			Healthy control	χ^2^/*F*^1^	*p*-value
	Mild group	Moderate group	Severe group			
*n*	20	20	20	20		
Sex (*n*%, male)	9 (45)	14 (70)	15 (75)	12(60)	4.45^1^	0.108
Age (years)	65.06 ± 7.83	67.81 ± 6.40	62.35 ± 7.7	62.26 ± 8.14	2.09^2^	0.110
Education (years)	8.6 ± 1.83	9.2 ± 0.23	8.8 ± 0.85	8.5 ± 0.53	3.74^2^	0.637
CS (*n*, %)	4 (20)	10 (50)	12 (60.0)	5 (25)	7.06^1^	0.029*
CAU (*n*, %)	2 (10)	8 (40)	9 (45)	3 (15.0)	6.62^1^	0.036*
HBP (*n*, %)	6 (30)	15 (75)	7 (35)	8 (40.0)	9.78^1^	0.008*
DM (*n*, %)	5 (25)	7 (35)	4 (20)	4 (20.0)	1.19^1^	0.5501
TC	4.67 ± 0.68	4.106 ± 0.86	3.57 ± 0.99	4.39 ± 0.96	5.43^2^	0.002*
TG	1.80 ± 1.82	1.58 ± 0.72	1.29 ± 0.57	1.45 ± 0.68	0.77^2^	0.516
HDL-C	1.23 ± 0.34	1.0275 ± 0.25	1.0155 ± 0.252	1.20 ± 0.31	2.71^2^	0.0512
LDL-C	2.60 ± 0.68	2.7300 ± 1.48	1.9605 ± 0.61	2.38 ± 0.72	2.50^2^	0.067
HCY	11.13 ± 2.74	16.87 ± 10.51	12.54 ± 3.23	12.59 ± 5.07	2.82^2^	0.045*
UA	327.07 ± 84.04	368.163 ± 79.51	296.495 ± 80.24	318.85 ± 59.87	2.69^2^	0.053
HAMD	18.53 ± 7.31	15.13 ± 9.60	15.55 ± 7.58	11.37 ± 7.68	2.42^2^	0.074
MMSE	24.94 ± 2.28	24.81 ± 2.17	23.25 ± 3.04	28.26 ± 1.046	16.6^2^	0.003*
TMT	24.06 ± 1.35	23.50 ± 4.49	22.75 ± 2.31	24.74 ± 0.93	2.08^2^	0.111
DST (forward)	5.76 ± 0.66	5.63 ± 0.72	5.65 ± 01.14	6.05 ± 0.41	1.15^2^	0.337
DST (backword)	4.53 ± 0.72	4.75 ± 0.56	3.95 ± 0.76	5.32 ± 0.67	13.05^2^	0.000*
RVR	26.71 ± 0.99	22.00 ± 1.75	22.30 ± 2.578	26.00 ± 1.94	28.17^2^	0.000*
CDT	3.06 ± 0.243	3.00 ± 0.52	3.15 ± 0.67	3.42 ± 0.51	2.35^2^	0.080

Continuous variables were presented as the means ± standard deviation. Categorical variables were presented as percentage. Chi-square test and one-way ANOVA were used to determine significant differences of the frequencies of categories and differences in continuous variables, respectively, between the groups. CS, current smoking; CAU, current alcohol use, HBP, high blood pressure; DM, diabetes mellitus; TC, total cholesterol; TG, triglyceride; LDL-C, low-density lipoprotein cholesterol; HDL-C, high-density lipoprotein cholesterol; HCY, homocysteine; UA, uric acid; HAMD, Hamilton Depression Scale; MMSE, Mini-Mental State Examination; TMT, Trail-Making Test; DST, Digital Span Test; RVR, Rapid Verbal Retrieve; CDT, Clock Drawing Test. ^1,2,*^: Chi-square test, one-way ANOVA, and *p* < 0.05, respectively.

The clinical characteristics of the asymptomatic patients with right carotid artery stenosis and healthy controls are shown in Table 2. There was no significant difference in age, sex and education nor in vascular risk factors examined in the study. However, Table 2 demonstrates that the scores of all the cognitive tests, including MMSE, TMT, DST, and CDT (*p* < 0.05), in patients with right CAS showed statistically significant differences when compared with healthy controls. These results revealed that many more special cognitive domains were impaired in asymptomatic patients with right CAS, including memory, language, attention, executive function and visuospatial function, than in those with left CAS.

**Table 2:** Differences of clinical risk factors and neuropsychological examinations of patients with right carotid artery stenosis.

Characteristic	Patients with right carotid artery stenosis			Healthy control	χ^2^/F	*p*-value
	Mild group	Moderate group	Severe group			
*n*	20	20	20	20		
Sex (*n*%, male)	11 (55)	16 (80.0)	16 (80.0)	12 (60.0)	4.829^1^	0.185
Age (years)	59.80 ± 9.17	62.00 ± 9.60	62.20 ± 8.59	62.25 ± 7.93	0.356^2^	0.785
Education (years)	9.1 ± 0.92	8.5 ± 0.68	8.6 ± 0.93	8.5 ± 0.53	7.365^2^	0.683
CS (*n*, %)	6 (30)	12 (60)	11 (55)	5 (25)	7.570^1^	0.056
CAU (*n*, %)	6 (30)	9 (45)	7 (35)	3 (15)	4.363^1^	0.225
HBP (*n*, %)	15 (75)	13 (65)	9 (45)	8 (40)	6.654^1^	0.084
DM (*n*, %)	3 (15)	7 (35)	6 (30)	4 (20)	2.667^1^	0.446
TC	4.19 ± 1.14	4.25 ± 1.47	3.84 ± 1.32	4.48 ± 1.01	0.905^2^	0.443
TG	1.35 ± 0.52	1.67 ± 1.06	1.95 ± 1.46	1.47 ± 0.67	1.428^2^	0.241
HDL-C	1.15 ± 0.35	1.04 ± 0.26	0.98 ± 0.23	1.20 ± 0.31	2.299^2^	0.084
LDL-C	2.55 ± 0.87	2.38 ± 1.10	1.95 ± 0.84	2.38 ± 0.72	1.643^2^	0.187
HCY	11.82 ± 4.76	12.53 ± 3.26	13.32 ± 6.86	12.31 ± 5.10	0.283^2^	0.838
UA	340.59 ± 76.92	347.83 ± 61.37	326.63 ± 81.89	318.74 ± 58.27	0.702^2^	0.554
HAMD	20.65 ± 8.98	19.50 ± 9.32	14.95 ± 8.76	11.75 ± 7.66	4.493^2^	0.006*
MMSE	26.05 ± 1.67	22.05 ± 5.65	22.70 ± 3.20	28.20 ± 1.06	14.520^2^	0.000*
TMT	24.50 ± 1.24	22.25 ± 2.221	22.65 ± 2.43	24.75 ± 0.91	9.772^2^	0.000*
DST (forward)	6.00 ± 0.65	5.45 ± 0.83	4.40 ± 0.75	6.05 ± 0.39	25.735^2^	0.000*
DST (backward)	5.25 ± 0.85	4.05 ± 0.83	3.90 ± 0.55	5.30 ± 0.66	21.194^2^	0.000*
RVR	25.30 ± 2.13	21.30 ± 2.18	20.70 ± 2.13	25.95 ± 1.91	33.287^2^	0.000*
CDT	3.20 ± 0.41	3.05 ± 0.67	2.80 ± 0.52	3.40 ± 0.50	4.389^2^	0.007*

Continuous variables were presented as the means ± standard deviation. Categorical variables were presented as percentage. Chi-square test and one-way ANOVA were used to determine significant differences of the frequencies of categories and differences in continuous variables, respectively, between the groups. CS, current smoking; CAU, current alcohol use, HBP, high blood pressure; DM, diabetes mellitus; TC, total cholesterol; TG, triglyceride; LDL-C, low-density lipoprotein cholesterol; HDL-C, high-density lipoprotein cholesterol; HCY, homocysteine; UA, uric acid; HAMD, Hamilton Depression Scale; MMSE, Mini-Mental State Examination; TMT, Trail-Making Test; DST, Digital Span Test; RVR, Rapid Verbal Retrieve; CDT, Clock Drawing Test. ^1,2,*^: Chi-square test, one-way ANOVA and *p* < 0.05, respectively.

### Pearson correlation analysis between vascular risk factors and impaired cognitive function

The correlative analysis between vascular risk factors and decreased cognitive function in asymptomatic patients with left CAS evaluated by the Pearson correlation coefficient is presented in Table 3. For these patients, there was a negative correlation between the degree of carotid stenosis and MMSE (*r* = −0.591, *p* = 0.000), DST (backward) (*r* = −0.544, *p* = 0.000), and RVR (*r* = −0.627, *p* = 0.000). In addition, there was a negative correlation between patient age and the MMSE (*r* = −0.240, *p* = 0.003) and RVR (*r* = −0.293, *p* = 0.008) scores. The MMSE scores were positively correlated with HDL-C (*r* = 0.244, *p* = 0.030) and negatively correlated with HAMD (*r* = −0.316, *p* = 0.004). The DST (backward) scores were negatively correlated with both TG (*r* = −0.248, *p* = 0.027) and HAMD (*r* = −0.496, *p* = 0.000). RVR scores were positively correlated with TC (*r* = 0.229, *p* = 0.041) and HDL-C (*r* = 0.300, *p* = 0.007) and negatively correlated with HCY (*r* = −0.301, *p* = 0.009).

**Table 3:** Pearson correlation analysis between vascular risk factors and impaired cognitive function in asymptomatic patients with left carotid artery stenosis.

Risk factors	*n*	MMSE		DST (backward)		RVR	
		*r*	*p* (two-tailed)	*r*	*p* (two-tailed)	*r*	*p* (two-tailed)
Age	80	−0.240	0.003*	−0.065	0.569	−0.293	0.008*
Sex	80	−0.122	0.282	0.081	0.473	−0.234	0.037*
Degree of stenosis	80	−0.591	0.000*	−0.544	0.000*	−0.627	0.000*
CS	80	−0.171	0.129	−0.099	0.382	−0.298	0.007*
CAU	80	−0.143	0.207	−0.132	0.243	−0.373	0.001*
HBP	80	0.041	0.721	0.056	0.623	−0.071	0.529
DM	80	0.064	0.570	0.078	0.491	−0.020	0.857
TC	80	0.114	0.312	−0.022	0.848	0.229	0.041*
TG	80	−0.107	0.347	−0.248	0.027*	−0.065	0.567
HDL-C	80	0.244	0.030*	0.091	0.424	0.300	0.007*
LDL-C	80	−0.027	0.816	0.087	0.445	0.044	0.697
HCY	80	−0.132	0.259	−0.001	0.994	−0.301	0.009*
UA	80	−0.089	0.433	0.061	0.592	−0.021	0.852

CS, current smoking; CAU, current alcohol use, HBP, high blood pressure; DM, diabetes mellitus; TC, total cholesterol; TG, triglyceride; LDL-C, low-density lipoprotein cholesterol; HDL-C, high-density lipoprotein cholesterol; HCY, homocysteine; UA, uric acid. *r*: Pearson correlation coefficients. *p < 0.05. *p*-values were obtained using the two-tailed Pearson correlation analysis.

Similarly, Table 4 shows the correlative analysis between vascular risk factors and decreased cognitive function in asymptomatic patients with right CAS evaluated by the Pearson correlation coefficient. As shown in Table 4, there was a negative correlation between patient age and all the cognitive scales. There was a negative correlation between the degree of carotid stenosis and MMSE (*r* = −0.675, *p* = 0.000), TMT (*r* = −0.520, *p* = 0.000), DST (forward) (*r* = −0.657, *p* = 0.000), DST (backward) (*r* = −0.641, *p* = 0.000), RVR (*r* = −0.730, *p* = 0.000) and CDT (*r* = −0.377, *p* = 0.001) for patients with right CAS. At the same time, there was a positive correlation with TC (*r* = 0.228, *p* = 0.042) and HDL-C (*r* = 0.224, *p* = 0.047) in the MMSE results.

**Table 4:** Pearson correlation analysis between vascular risk factors and impaired cognitive function in asymptomatic patients with right carotid artery stenosis.

Risk factors	*n*	MMSE		TMT		DST (forward)		DST (backward)		RVR		CDT	
		*r*	*p* (two-tailed)	*r*	*p* (two-tailed)	*r*	*p* (two-tailed)	*r*	*p* (two-tailed)	*r*	*p* (two-tailed)	*r*	*p* (two-tailed)
Age	80	−0.315	0.004*	−0.360	0.001*	−0.332	0.003*	−0.284	0.011*	−0.372	0.001*	−0.308	0.005*
Sex	80	−0.188	0.095	−0.154	0.174	−0.322	0.004*	−0.126	0.265	−0.338	0.002*	−0.197	0.080
Degree of stenosis	80	−0.675	0.000*	−0.520	0.000*	−0.657	0.000*	−0.641	0.000*	−0.730	0.000*	−0.377	0.001*
CS	80	−0.215	0.055	−0.218	0.052	−0.284	0.011*	−0.188	0.094	−0.264	0.018*	−0.119	0.294
CAU	80	−0.145	0.200	−0.101	0.374	−0.153	0.175	−0.103	0.364	−0.135	0.234	0.004	0.971
HBP	80	−0.051	0.653	−0.037	0.742	0.069	0.540	0.052	0.648	0.020	0.862	−0.056	0.624
DM	80	−0.145	0.198	−0.169	0.135	−0.139	0.218	−0.219	0.051	−0.170	0.132	−0.061	0.588
TC	80	0.228	0.042*	0.108	0.341	−0.031	0.783	0.118	0.298	0.029	0.800	−0.094	0.406
TG	80	0.030	0.790	0.055	0.625	−0.106	0.348	−0.040	0.725	−0.092	0.416	−0.094	0.407
HDL-C	80	0.224	0.047*	0.086	0.450	0.198	0.080	0.178	0.116	0.171	0.131	0.067	0.560
LDL-C	80	0.171	0.131	0.138	0.225	0.017	0.880	0.154	0.175	0.080	0.481	−0.116	0.309
HCY	80	−0.085	0.462	−0.212	0.062	−0.079	0.490	−0.180	0.114	−0.062	0.591	−0.081	0.480
UA	80	−0.146	0.196	0.100	0.377	−0.035	0.758	0.106	0.349	−0.011	0.923	0.073	0.522

CS, current smoking; CAU, current alcohol use, HBP, high blood pressure; DM, diabetes mellitus; TC, total cholesterol; TG, triglyceride; LDL-C, low-density lipoprotein cholesterol; HDL-C, high-density lipoprotein cholesterol; HCY, homocysteine; UA, uric acid. *r*: Pearson correlation coefficients. *p < 0.05. *p*-values were obtained using the two-tailed Pearson correlation analysis.

### Multiple logistic regression analysis for risk factors of cognitive impairment

Multiple logistic regression analyses of age, sex, degree of stenosis, CS, CAU, TC, TG, HDL-C, LDL-C, and HCY were performed to identify risk factors for cognitive impairment (Tables 5, 6). In patients with left CAS, the degree of stenosis was a risk factor for low MMSE (OR = 0.275, 95% CI 0.143−0.531), DST (backward) (OR = 0.355, 95% CI 0.204−0.618) and RVR (OR = 0.147, 95% CI 0.058−0.376) scores. Patient age was a risk factor for low MMSE (OR = 0.910, 95% CI 0.841−0.984) and RVR (OR = 0.881, 95% CI 0.792−0.979) scores (Table 5). Moreover, the degree of stenosis (OR = 0.147, 95% CI 0.058−0.376) was a risk factor for low DST (backward) scores in patients with left CAS (Table 5). As shown in Table 6, patient age was a risk factor for all cognitive scales except MMSE. The degree of stenosis was a risk factor for low TMT (OR = 0.247, 95% CI 0.128–0.475), DST (forward) (OR = 0.153, 95% CI 0.068–0.345), DST (backward) (OR = 0.122, 95% CI 0.049–0.306), RVR (OR = 0.068, 95% CI 0.020–0.228) and CDT (OR = 0.501, 95% CI 0.283–0.886) scores in patients with right CAS. Additionally, the degree of stenosis was also a risk factor for MMSE (OR = 0.249, 95% CI 0.127–0.490) in patients with right CAS (Table 6).

**Table 5:** Multiple logistic regression analysis for risk factors of cognitive impairment in patients with left carotid artery stenosis.

Risk factors	*B* coefficient	Std. error	*p*	OR	95% CI	
					Upper	Lower
MMSE						
Age	−0.095	0.040	0.018*	0.910	0.841	0.984
Degree of stenosis	−1.289	0.335	0.000*	0.275	0.143	0.531
HDL-C	0.323	1.076	0.764	1.382	0.168	11.382
DST (backward)						
Degree of stenosis	−1.036	0.283	0.000*	0.355	0.204	0.618
TG	−0.294	0.279	0.293	0.746	0.431	1.289
RVR						
Age	−0.127	0.054	0.019*	0.881	0.792	0.979
Sex	1.744	1.084	0.108	5.720	0.683	47.910
Degree of stenosis	−1.915	0.478	0.000*	0.147	0.058	0.376
CS	−0.736	1.168	0.528	0.479	0.049	4.724
CAU	−0.196	1.214	0.872	0.822	0.076	8.880
TC	0.089	0.507	0.860	1.093	0.405	2.950
HDL-C	0.219	1.545	0.887	1.244	0.060	25.731
HCY	−0.218	0.135	0.106	0.804	0.617	1.047

CS, current smoking; CAU, current alcohol use, TC, total cholesterol; TG, triglyceride; HDL-C, high-density lipoprotein cholesterol; HCY, homocysteine. OR, Odds ratio. *p < 0.05.

**Table 6:** Multiple logistic regression analysis for risk factors of cognitive impairment in patients with right carotid artery stenosis.

Risk factors	*B* coefficient	Std. error	*p*	OR	95% CI	
					Upper	Lower
MMSE						
Age	−0.051	0.036	0.153	0.950	0.885	1.019
Degree of stenosis	−1.390	0.345	0.000*	0.249	0.127	0.490
TC	0.199	0.258	0.441	1.220	0.735	2.024
HDL-C	−0.196	1.131	0.863	0.822	0.090	7.546
TMT						
Age	−0.116	0.039	0.003*	0.890	0.825	0.960
Degree of stenosis	−1.400	0.335	0.000*	0.247	0.128	0.475
DST (forward)						
Age	−0.093	0.039	0.017*	0.912	0.845	0.984
Sex	−1.704	0.987	0.084	0.182	0.026	1.259
Degree of stenosis	−1.878	0.416	0.000*	0.153	0.068	0.345
CS	0.408	0.825	0.621	1.504	0.298	7.583
DST (backward)						
Age	−0.115	0.042	0.007*	0.892	0.821	0.969
Degree of stenosis	−2.103	0.469	0.000*	0.122	0.049	0.306
RVR						
Age	−0.203	0.056	0.000*	0.816	0.731	0.912
Sex	−1.175	1.115	0.292	0.309	0.035	2.746
Degree of stenosis	−2.687	0.616	0.000*	0.068	0.020	0.228
CS	0.596	1.060	0.574	1.815	0.227	14.482
CDT						
Age	−0.099	0.039	0.010*	0.906	0.840	0.977
Degree of stenosis	−0.691	0.291	0.018*	0.501	0.283	0.886

CS, current smoking; TC, total cholesterol; HDL-C, high-density lipoprotein cholesterol. OR, odds ratio. *p < 0.05.

### Receiver operating characteristic curve for independent risk factors of cognitive impairment

Table 7 presents the independent risk factors for cognitive impairment in asymptomatic patients with unilateral CAS that were evaluated by ROC curve analysis. In the patients with left CAS, degree of stenosis was an independent risk factor for low MMSE (AUC = 0.194, *p* = 0.000), DST (backward) (AUC = 0.056, *p* = 0.000) and RVR (AUC = 0.129, *p* = 0.000) scores. Patient age was an independent risk factor for low MMSE scores (AUC = 0.355, *p* = 0.025). In the patients with right CAS, degree of stenosis was an independent risk factor for low MMSE (AUC = 0.172, *p* = 0.000), TMT (AUC = 0.190, *p* = 0.000), DST (forward) (AUC = 0.129, *p* = 0.000), DST (backward) (AUC = 0.134, *p* = 0.000), RVR (AUC = 0.112, *p* = 0.039) and CDT (AUC = 0.321, *p* = 0.021) scores. Patient age was an independent risk factor for all cognitive scale scores except the MMSE score (Table 7). Thus, these data revealed that the degree of stenosis was an independent risk factor for impaired cognition in asymptomatic patients with CAS.

**Table 7:** The receiver operating characteristic curve performed for independent risk factors of cognitive impairment in patients with carotid artery stenosis.

Risk factors	AUC	Std. error	*p*	95% CI	
				Upper	Lower
Patients with left carotid artery stenosis					
MMSE					
Age	0.355	0.063	0.025*	0.478	0.232
Degree of stenosis	0.194	0.049	0.000*	0.289	0.098
DST (backward)					
Degree of stenosis	0.244	0.056	0.000*	0.353	0.134
TG	0.523	0.070	0.735	0.660	0.386
RVR					
Age	0.386	0.063	0.079	0.510	0.261
Degree of stenosis	0.129	0.046	0.000*	0.220	0.038
Patients with right carotid artery stenosis					
MMSE					
Degree of stenosis	0.172	0.045	0.000*	0.261	0.084
TMT					
Age	0.300	0.060	0.003*	0.420	0.180
Degree of stenosis	0.190	0.050	0.000*	0.290	0.100
DST (forward)					
Age	0.347	0.062	0.020*	0.469	0.225
Degree of stenosis	0.129	0.040	0.000*	0.208	0.050
DST (backward)					
Age	0.351	0.062	0.023*	0.473	0.230
Degree of stenosis	0.134	0.043	0.000*	0.217	0.050
RVR					
Age	0.266	0.057	0.000*	0.378	0.154
Degree of stenosis	0.112	0.039	0.000*	0.187	0.036
CDT					
Age	0.299	0.071	0.010*	0.439	0.159
Degree of stenosis	0.321	0.068	0.021*	0.455	0.187

TG, triglyceride. AUC: area under curve. *p < 0.05.

### Voxel-based morphometry analysis of brain area volumes

**Figure 1:**
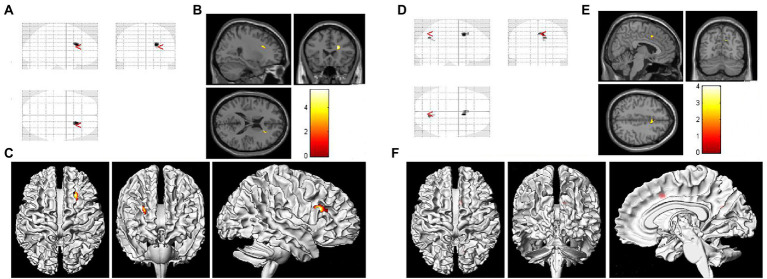
Voxel-based morphometry (VBM) results of gray matter atrophy and white matter lesions in asymptomatic patients with right severe carotid artery stenosis compared with healthy controls. **(A,D)** Statistical parametric mapping (SPM) results in various brain regions. **(B)** Significantly decreased white matter volume was found in the right subgyral region (*p* < 0.05). **(E)** Gray matter atrophy was found in the right occipital lobe precuneus and right occipital lobe cuneus (*p* < 0.05). **(C,F)** Three-dimensional images of the asymptomatic patient with right severe carotid artery stenosis.

Figure 1 showed the VBM results for gray matter atrophy and white matter lesions in asymptomatic patients with right severe carotid artery stenosis compared with those in healthy controls. The red or yellow spots in Figure 1 indicated the brain regions with abnormal volumes in patients with right severe carotid artery stenosis. The gray and white matter volumes based on VBM analysis were compared between the patients with unilateral CAS and healthy controls by independent-sample *t-test*s. Table 8 shows that the gray matter volumes of three areas, left parietal lobe precuneus (*T* = 2.08, *p* < 0.05), right anterior cingulate (*T* = 2.82, *p* < 0.05), and right cingulate gyrus (*T* = 4.78, *p* < 0.05), obviously declined in the patients with left severe CAS when compared with healthy controls. Significantly decreased gray matter volumes were detected not only in the right parahippocampal gyrus (*T* = 1.93, *p* < 0.05) but also in the left supplementary motor area (*T* = 3.32, *p* < 0.05) in patients with right moderate CAS compared with healthy controls. Finally, there was a significant decrease in gray matter volume in the right occipital lobe precuneus (*T* = 2.06, *p* < 0.05) and right occipital lobe cuneus (*T* = 2.98, *p* < 0.05) in patients with right severe CAS compared with healthy controls. As shown in Table 9, significantly decreased white matter volume was detected in the left subgyral region in the patients with left severe CAS compared with healthy controls (*T* = 3.85, *p* < 0.05). There was a significant decline in left insula white matter volume in the patients with right moderate CAS compared with healthy controls (*T* = 3.63, *p* < 0.05). Additionally, white matter volume in the right subgyral region in the patients with right severe CAS was significantly decreased compared with that in the healthy controls (*T* = 5.47, *p* < 0.05).

**Table 8:** Voxel-based morphometry (VBM) analysis of the declined gray matter volume in patients with carotid artery stenosis compared with healthy controls.

Groups	Gray matter				
	Brain area	*T* value	MNI coordinate		
			*x*	*y*	*z*
Group with left severe carotid stenosis *vs.* healthy controls	Left parietal lobe//Precuneus	2.08	−9.0	−70.5	24.0
	Right limbic lobe//Anterior cingulate	2.82	9.0	22.5	27.0
	Right frontal lobe//Cingulate gyrus	4.78	6.0	10.5	40.5
Group with right moderate carotid stenosis *vs.* healthy controls	Left limbic lobe//Parahippocampal gyrus	1.93	−27.0	−18.0	−16.5
	Supp_Motor_Area_L	3.32	−0.0	19.5	45.0
Group with right severe carotid stenosis *vs.* healthy controls	Right occipital lobe//Precuneus	2.06	10.5	−61.5	27.0
	Right occipital lobe//Cuneus	2.98	12.0	−70.5	31.5

**Table 9:** Voxel-based morphometry analysis of the declined white matter volume in patients with carotid artery stenosis compared with healthy controls.

Groups	White matter				
	Brain area	*T* value	MNI coordinate		
			*x*	*y*	*z*
Group with left severe carotid stenosis *vs.* healthy controls	Left temporal lobe/Sub-Gyral	3.85	13.5	−67.5	−39.0
Group with right moderate carotid stenosis *vs.* healthy controls	Left sub-lobar/Insula	3.63	−34.5	−9.0	22.5
					
Group with right severe carotid stenosis *vs.* healthy controls	Right frontal lobe/Sub-gyral	5.47	22.5	22.5	22.5

## Discussion

Carotid artery atherosclerotic stenosis increases the risk of ischemic stroke, and the estimated rate of ipsilateral carotid-related acute ischemic stroke is 4.7% over 5 years (Balestrini et al., 2013; Chang et al., 2022). Increasing evidence has demonstrated that cognitive impairment is also associated with asymptomatic bilateral and unilateral CAS (Cheng et al., 2012; Xiang et al., 2013; Zavoreo et al., 2013; Buratti et al., 2014). Consistent with previous studies, our results revealed that unilateral CAS can contribute to cognitive decline. More importantly, many more cognitive domains were impaired in the patients with right CAS than in those with left CAS. Cognitive impairments found in asymptomatic patients with right CAS included those in the memory, language, attention, executive and visuospatial functions. These cognitive functions are also impaired as a result of vascular cognitive impairment (Bogolepova, 2022; Boomsma et al., 2022). Thus, our results indicated that CAS, especially right CAS, might play an important role in the development of vascular cognitive impairment. Additionally, multiple logistic regression analysis and the ROC curve analysis were used to confirm whether CAS is an important contributor to cognitive decline. The results of statistical analysis revealed that the degree of carotid stenosis was an independent risk factor for cognitive impairment. Our results suggest that, for the asymptomatic patients with CAS, revascularization could not only relieve stenosis of the carotid artery, but also, to a certain extent, improve the cognitive impairment. This finding was in line with those of several recent studies (Gupta et al., 2020; Turowicz et al., 2021).

The underlying mechanism of the cognitive impairment induced by unilateral CAS remains unclear. Previous studies have speculated on the involvement of cerebral hypoperfusion caused by hemodynamic disorders (Kaczmarz et al., 2021) and the uncoupling of cerebral hemodynamic and metabolic states (Gottler et al., 2019). Recently, thinning of the cortex was considered a potential biomarker, as it was previously shown to be associated with cognitive impairment in aging, neurodegenerative disease, and small vascular disease (Pettigrew et al., 2016; Weston et al., 2016). Increasing evidence has shown that gray matter atrophy is correlated with cognitive decline measured in asymptomatic patients with CAS (Benli et al., 2021; Gao et al., 2021; Wang et al., 2021). In contrast, a significant reduction in cortical thickness was not found in brain regions ipsilateral to the carotid stenosis (Nickel et al., 2019). Whether gray matter atrophy plays an important role in cognitive decline in patients with CAS is currently still under debate. In the present study, the T1-weighted MRI images of the recruited patients were acquired and processed by VBM analysis using the VBM 8 toolbox for SPM 8 software in MATLAB 2012a. In line with a previous study (Wang et al., 2021), loss of gray matter volume in patients was not only limited to the hemisphere ipsilateral to the stenosis but also observed in the contralateral hemisphere. We found that compared with healthy controls, gray matter volumes in patients with left severe CAS were significantly lower, and gray matter atrophy was widely distributed in the left precuneus, right anterior cingulate, and right cingulate gyrus. Furthermore, the gray matter volumes in patients with both moderate and severe right CAS were significantly decreased compared with those in healthy controls, and gray matter atrophy was detected in the left parahippocampal gyrus, right precuneus and right cuneus. Our findings indicated that gray matter atrophy was particularly vulnerable to stenosis of the right carotid artery.

In addition, we analyzed and compared the white matter volume between patients with CAS and healthy controls. Our results showed that white matter volume significantly decreased in asymptomatic patients with CAS compared with healthy controls. Consistent with the results of gray matter volume analysis, markedly decreased white matter volume was found in patients not only with left CAS but also with right CAS. Moreover, white matter volume in the left insula and right subgyral regions was significantly reduced in patients with moderate and severe right CAS compared with healthy controls. Chronic cerebral hypoperfusion due to carotid stenosis or occlusion has been shown to cause white matter injuries in animal experiments (Washida et al., 2019). Recently, increasing evidence shows that white matter hyperintensities are also present in patients with CAS (Ye et al., 2018; Benli et al., 2021; Gao et al., 2021). Cerebral hypoperfusion might be an important contributor to white matter hyperintensities induced by carotid stenosis, and the completeness of collateral circulation could protect these patients against white matter hyperintensities (Ye et al., 2019). It has also been reported that the development of cognitive dysfunction might be associated with the destructive effect of white matter hyperintensities on brain functional connectivity in patients with CAS (Porcu et al., 2020a,b).

Finally, our findings also revealed that there was a positive correlation between the level of TC and the scores of the MMSE and RVR tests. High levels of serum cholesterol could alleviate the cognitive dysfunction, especially language and executive function. Previous studies have shown that a high level of serum cholesterol is positively correlated with an increased risk of dementia (Loera-Valencia et al., 2019), and some studies have reported a decreased prevalence of Alzheimer’s dementia in patients taking cholesterol-lowering drugs (Barone et al., 2014). In contrast, a meta-analysis showed that there was no clear consistent relationship between cholesterol and cognitive decline (Peters et al., 2021). Furthermore, some studies have demonstrated that a higher concentration of TC might be a protective factor for cognitive performance (Lv et al., 2016; Pang et al., 2022). Therefore, further experiments are needed to identify the correlation between serum TC and cognitive impairment.

## Limitation

Our study had a relatively small sample size and was a cross-sectional experiment based in a hospital. A specifically designed, randomized, controlled prospective population-based study is warranted in the future. Additionally, although we found abnormal brain regions in patients with CAS based on VBM analysis, we did not confirm the correlation between abnormal brain areas and impaired cognitive function. Finally, we did not explore the formation mechanism of abnormal brain areas caused by unilateral CAS.

## Conclusion

The current study provided insights into the association between cognitive impairment and carotid artery stenosis. Unilateral asymptomatic carotid artery stenosis, especially of the right carotid artery, was significantly related to cognitive impairment, including memory, language, attention, executive function and visuospatial function. More importantly, both gray matter and white matter volumes detected by VBM analysis significantly declined in patients with unilateral asymptomatic carotid artery stenosis. The degree of carotid stenosis was an independent risk factor for cognitive impairment. Revascularization might prevent cognitive dysfunction in patients. In addition, there was a positive correlation with TC and special cognitive domains. The lower the cholesterol level is, the more severe the cognitive impairment.

## Data availability statement

The raw data supporting the conclusions of this article will be made available by the authors, without undue reservation.

## Author contributions

DD: conception and design, final approval of the version to be published. WD: participation in the whole work, drafting of the manuscript, and data analysis. CC and TS: MRI data acquisition and assessment. LL: demographic and cardiovascular risk factor data collection. All authors contributed to the article and approved the submitted version.

## Funding

This work was supported by the Program of the National Natural Science Foundation of China (81873757) and Natural Science Foundation Project of Chongqing (cstc2021jcyj-msxmX0494) and Special Project for Scientific and Technological Innovation of Social Undertakings and Livelihood Security in Chongqing (cstc2017shmsA130004).

## Conflict of interest

The authors declare that the research was conducted in the absence of any commercial or financial relationships that could be construed as a potential conflict of interest.

## Publisher’s note

All claims expressed in this article are solely those of the authors and do not necessarily represent those of their affiliated organizations, or those of the publisher, the editors and the reviewers. Any product that may be evaluated in this article, or claim that may be made by its manufacturer, is not guaranteed or endorsed by the publisher.

## Footnotes

^1^ http://dbm.neuro.uni-jena.de/vbm.html
